# Analytic modeling of neural tissue: II. Nonlinear membrane dynamics

**DOI:** 10.1063/5.0124414

**Published:** 2022-11-14

**Authors:** B. L. Schwartz, S. M. Brown, J. Muthuswamy, R. J. Sadleir

**Affiliations:** 1 School of Biological and Health Systems Engineering, Arizona State University, 501 E Tyler Mall, Tempe, Arizona 85287-9709, USA; 2 Department of Physical Sciences, Phoenix College, 1202 W Thomas Road, Phoenix, Arizona 85013-4208, USA; 3 School of Computing, Informatics, and Decision Systems Engineering, Arizona State University, 699 S Mill Avenue, Tempe, Arizona 85281-3636, USA

## Abstract

Computational modeling of neuroactivity plays a central role in our effort to understand brain dynamics in the advancements of neural engineering such as deep brain stimulation, neuroprosthetics, and magnetic resonance electrical impedance tomography. However, analytic solutions do not capture the fundamental nonlinear behavior of an action potential. What is needed is a method that is not constrained to only linearized models of neural tissue. Therefore, the objective of this study is to establish a robust, straightforward process for modeling neurodynamic phenomena, which preserves their nonlinear features. To address this, we turn to decomposition methods from homotopy analysis, which have emerged in recent decades as powerful tools for solving nonlinear differential equations. We solve the nonlinear ordinary differential equations of three landmark models of neural conduction—Ermentrout–Kopell, FitzHugh–Nagumo, and Hindmarsh–Rose models—using George Adomian’s decomposition method. For each variable, we construct a power series solution equivalent to a generalized Taylor series expanded about a function. The first term of the decomposition series comes from the models’ initial conditions. All subsequent terms are recursively determined from the first. We show rapid convergence, achieving a maximal error of $<10^{-12}$ with only eight terms. We extend the region of convergence with one-step analytic continuation so that our complete solutions are decomposition splines. We show that this process can yield solutions for single- and multi-variable models and can characterize a single action potential or complex bursting patterns. Finally, we show that the accuracy of this decomposition approach favorably compares to an established polynomial method, B-spline collocation. The strength of this method, besides its stability and ease of computation, is that, unlike perturbation, we make no changes to the models’ equations; thus, our solutions are to the problems at hand, not simplified versions. This work validates decomposition as a viable technique for advanced neural engineering studies.

## I. INTRODUCTION

New advanced MRI techniques allow for *in vivo* quantitative inquiries of properties of tissue by perturbing it in synchrony with the imaging pulse sequence such that its response is encoded in the phase component of the complex magnetic resonance (MR) signal.^1^ From the phase map, we can then calculate the key parameters that can serve as biomarkers for tissue health. For example, in brain MR elastography, the tissue is vibrated, and the mechanical shear waves are encoded in the phase maps from which we can determine the tissue’s complex shear modulus.^2^ In MR electrical impedance tomography (MREIT), the perturbation is injected electric current whose density **J**(**r**) throughout the tissue is determined by the distributed electrical conductivity ***σ***(**r**). The phase contrast encodes the magnetic field **B**(**r**) arising from **J**(**r**), whence we can determine ***σ***(**r**).^3^ Sadleir *et al.* proposed^4^ and experimentally demonstrated^5^ using MREIT to detect neural activity directly, exploring the conductivity changes that result from active membrane dynamics. It is therefore appropriate to focus our analytic power on models of the active membrane, which are highly nonlinear.

Most biologic—indeed, most engineering—phenomena are characterized by nonlinear differential equations, which most analytic methods cannot solve directly but instead must have the equations simplified through, e.g., linearization or dramatically restricting the parameter space, that is, the solutions are to problems different from the original modeling, curtailing their applicability. Sometimes such changes are perfectly sensible because the nonlinear behavior is not the salient detail under scrutiny, such as Rall and Agmon-Snir using the cable equation to model neurons to consider impulse transmission along large distances^6^ or how in the first study of this series Schwartz *et al.* held the membrane as purely resistive to consider a snapshot of electromagnetic field distribution throughout a bidomain.^7^ In this study, we will look at three increasingly complex models of membrane conduction. The first is the theta model by Ermentrout and Kopell.^8,9^ Using only one variable, this quadratic integrate and fire model describes bursting in *Aplysia* neurons. Next we consider the model by Fitzhugh^10^ and Nagumo *et al.*,^11^ which shows the all-or-nothing phenomenon of an action potential once the transmembrane voltage has surpassed a certain threshold. The final model is by Hindmarsh and Rose^12^ and adds a third variable that allows for the description of oscillatory burst discharge. The nonlinear nature of these models is key to the frontier problems of neural engineering, e.g., MREIT; hence, our solution method must include it.

Over the last 30 or so years, a rich scholarship comprising the broad field of homotopy analysis has been developed^13^ with the goal of a unified method for solving all sorts of mathematic models of the natural world—linear, nonlinear, deterministic, and stochastic—and which remains largely unexplored in the neural engineering community. Broadly, these techniques are recursive, starting with the initial or boundary conditions and creating an analytic series solution. Specifically in this work, we will use the decomposition method developed by Adomian^14^ but with modifications added later, which we will note. For convenience, we will simply refer to it as the Adomian decomposition method (ADM). The ADM creates a power series solution, called a decomposition series, which is equivalent to a Taylor series, by decomposing the unknown function into an infinite series, defining the first term as the initial condition. The nonlinear components are decomposed into their own infinite series called Adomian polynomials, which also start with the initial condition. Each term in the decomposition series depends on the one before it; hence, starting from the initial conditions, all terms are recursively calculated. The reviews by Adomian^15^ and Rach^16^ are both excellent primers on this method.

The ADM has gained some traction in the realms of biomedical phenomena, having been applied to nonlinear models of cellular population growth,^17^ cellular systems and aging,^18^ and infection diseases and immune response.^19^ Adomian^20^ and Adomian *et al.*^21^ discussed a limited time-and-space-dependent version of the FitzHugh–Nagumo model, which had only one state variable, as well as the Hodgkin–Huxley model,^21^ to describe impulse propagation down an axon. These studies are largely theoretic, serving more as proposals, without worked concrete examples, error analysis, or analytic continuation, all of which we address here. Furthermore, we will only consider models described by ordinary differential equations, neglecting spatial dependence.

We will start with a brief description of the ADM in Sec. II, continue with detailed analyses of our three models in Sec. III, compare the Adomian spline solutions to cardinal basis spline solutions in Sec. IV, and end with remarks on future directions.

## II. MATHEMATICAL PRELIMINARIES

### A. Operator notation

Consider a heterogeneous nonlinear ordinary differential equation F{u(t)}=g(t) whose operator F is the sum of linear L and R and nonlinear N terms,

F{u}=L{u}+R{u}+N{u}=g, (1)

where $L=\frac{d^{n}}{dt^{n}}$ is the highest order differential operator, which we assume to be easily invertible, R is the remaining part(s) of the linear operation, N is the nonlinear operator, and *g* is a given function. Hereafter, we will neglect the curly brackets around arguments with only one variable. We assume that $L^{-1}Lu=u-\Phi$, where $L^{-1}$ is the *n*-fold definite integral operator from 0 to *t*, whence we determine Φ by the initial value(s). Thus, if $L=\frac{d}{dt}$, then $L^{-1}=\int_{0}^{t}dt$ and Φ = *u*(0). Applying $L^{-1}$ through (1) and solving for *u* yield

$$u=\Phi +L^{-1}g-L^{-1}Ru-L^{-1}Nu.$$ (2)

### B. Decomposition series

The ADM assumes that we can decompose the solution into an infinite series,^14^

$$u=\overset{\infty }{\underset{n=0}{\sum }}u_{n},$$ (3)

with the first term coming from the initial condition. The nonlinear terms Nu are themselves decomposed into the Adomian polynomials,

$$Nu=\overset{\infty }{\underset{n=0}{\sum }}A_{n},$$ (4)

which are defined as^22^

$$A_{n}={\left.\frac{1}{n!}\frac{d^{n}}{d\zeta^{n}}N\left(\overset{\infty }{\underset{n=0}{\sum }}u_{n}\zeta^{n}\right)\right|}_{\zeta =0}.$$ (5)

See Appendix A for an example of the Mathematica code for generating *A*_*n*_. The components of *u* are determined from the recursion scheme,^23^

$$\begin{array}{c} u_{0}=\Phi ,\\ u_{1}=L^{-1}g-L^{-1}Ru_{0}-L^{-1}A_{0},\\ u_{n+2}=-L^{-1}Ru_{n+1}-L^{-1}A_{n+1}. \end{array}$$ (6)

From Eq. (5), we see that *A*_0_ comes from the initial condition *u*_0_, *A*_1_ from *u*_0_ and *u*_1_, *A*_2_ from *u*_0_, *u*_1_, and *u*_2_, and so on. Thus, all components in Eq. (3) are determined. The solution converges quickly,^24^ so in practice, the *m*-term partial sum will suffice, given here as

$$\varphi_{m}\left(t\right)=\overset{m-1}{\underset{n=0}{\sum }}u_{n}\left(t\right);\underset{m\to \infty }{\lim}\;\varphi_{m}\left(t\right)=u\left(t\right).$$ (7)

### C. One-step analytic continuation

The decomposition series *φ*_*m*_(*t*) is equivalent to a generalized Taylor series^25^ about the function *u*_0_(*t*)^26^ and whose radius of convergence *r* is insufficient to characterize, e.g., rapidly varying neural dynamics.^27^ The solution^28,29^ to this shortcoming—built into the ADM itself—is a self-starting, simple to use, and accurate one-step method of analytic continuation of *φ*_*m*_(*t*) in terms of its family of elemental functions $\varphi_{m}^{\left(k\right)}\left(t\right)$ with overlapping *r*^(*k*)^,

$$\begin{array}{c} \varphi_{m}\left(t\right)=\varphi_{m}^{\left(0\right)}\left(t\right);t_{0}-r^{\left(0\right)}<t<t_{0}+r^{\left(0\right)},\\ \varphi_{m}\left(t\right)=\varphi_{m}^{\left(1\right)}\left(t\right);t_{1}-r^{\left(1\right)}<t<t_{1}+r^{\left(1\right)},\\ \vdots \\ \varphi_{m}\left(t\right)=\varphi_{m}^{\left(k\right)}\left(t\right);t_{k}-r^{\left(k\right)}<t<t_{k}+r^{\left(k\right)}. \end{array}$$ (8)

The domain of interest is partitioned into (variable) time intervals [0, *t*_1_), [*t*_1_, *t*_2_), …, [*t*_*k*−1_, *t*_*k*_), each with its analytic continuant $\varphi_{m}^{\left(k\right)}\left(t\right)$ of primitive $\varphi_{m}^{\left(0\right)}\left(t\right)$. From the condition of continuity $\varphi_{m}^{\left(k+1\right)}\left(t_{k+1}\right)=\varphi_{m}^{\left(k\right)}\left(t_{k+1}\right)$, the values at the end of each element are the initial conditions for the following element.^27^ The resultant spline of *N* + 1 elements is conveniently expressed as^30^

$$\varphi_{m}\left(t\right)=\overset{N}{\underset{k=0}{\sum }}\varphi_{m}^{\left(k\right)}\left(t\right)\Pi \left(t;t_{k},t_{k+1}\right),$$ (9)

where Π is the boxcar function.^31^ Appendix B shows our code for generating the spline elements. Neither the intervals’ durations *h*_*k*_ = *t*_*k*+1_ − *t*_*k*_ nor orders *m* of their partial sums need to be the same.^28,29^ Indeed, the spline can be optimized by (1), fixing each *m* while varying the intervals’ duration from *h*_*k*_ = *λr*^(*k*)^ where the dilation constant *λ* < 1 and the radii of convergence are estimated by the *m*th coefficient of each $\varphi_{m}^{\left(k\right)}\left(t\right)$ as $r_{m}^{\left(k\right)}={\sqrt[{m}]{\left|a_{m}^{\left(k\right)}\right|}}^{-1}$,^32^ or by (2), fixing *h*_*k*_ = *h* while varying *m* to be the smallest required to bring the interval’s maximum truncation error $\varepsilon^{\left(k\right)}=\max_{t_{k}\leq t\leq t_{k+1}}|\varphi_{m}^{\left(k\right)}\left(t\right)-\varphi_{m+1}^{\left(k\right)}\left(t\right)|$ below a set tolerance *ɛ*_tol_, or both.^33^

## III. ACTIVE MEMBRANE MODELS

### A. Ermentrout–Kopell model

Let us begin with a single variable model from Ermentrout and Kopell^8,9^ that can describe parabolic bursting behavior in *Aplysia* neurons. The theta model, as it is also known, is canonic for type I membrane dynamics—characterized by a wide range of firing frequencies^34^—because all other type I models can be reduced thereto^35,36^ and is given as

$$\begin{array}{c} \frac{d\theta }{dt}=q\left(1-\cos\left(\theta \right)\right)+\left(1+\cos\left(\theta \right)\right)\eta ,\\ \theta \in \left[0,2\pi \right],\theta \left(0\right)=\theta \left(2\pi \right)=0, \end{array}$$ (10)

where *θ* is the voltage, the constant *η* represents the input current, and *q* is the membrane time constant, which we can set to unity without loss of generality. When *η* > 0, via quadrature, the analytic solution in closed form is found to be^34^

$$\theta \left(t\right)=2\;\arctan\left(\sqrt{\eta }\;\tan\left(\sqrt{\eta }t\right)\right).$$ (11)

To approach the *θ* model with the ADM, let us re-arrange Eq. (10), conveniently isolating the nonlinear component,

$$\frac{d\theta }{dt}=1+\eta +\left(\eta -1\right)\cos\left(\theta \right).$$ (12)

We see that our operators are $L\theta =\frac{d\theta }{dt}$, Nθ=cos(θ), *g* = 1 + *η*, and R=0, so Eq. (12) becomes

Lθ=g+(η−1)Nθ. (13)

We write $\theta =\sum_{n=0}^{\infty }\theta_{n}$ and $N\theta =\sum_{n=0}^{\infty }A_{n}\left(\cos\left(\theta \right)\right)$, and, after applying $L^{-1}=\int_{0}^{t}dt$ throughout, Eq. (13) becomes

$$\theta \left(t\right)=\overset{\infty }{\underset{n=0}{\sum }}\theta_{n}=\theta \left(0\right)+\left(1+\eta \right)t+\left(\eta -1\right)\overset{\infty }{\underset{n=0}{\sum }}L^{-1}A_{n}\left(\cos\left(\theta \right)\right).$$ (14)

From Eq. (6), each *θ*_*n*_ is found from our recursion scheme,

$$\begin{array}{c} \theta_{0}=\theta \left(0\right),\\ \theta_{1}=\left.\left(1+\eta \right)t+\left(\eta -1\right)\right)L^{-1}A_{0}\left(\cos\left(\theta \right)\right),\\ \theta_{n+2}=\left(\eta -1\right)L^{-1}A_{n+1}\left(\cos\left(\theta \right)\right). \end{array}$$ (15)

Thus, our decomposed *θ*(*t*) is fully determined, completing our analysis. Because this model has a known analytic solution in closed form, we can easily verify our solution by hand. The first few $A_{n}\left(\cos\left(\theta \right)\right)$ values are

$$\begin{array}{c} A_{0}=\cos\left(\theta_{0}\right),\\ A_{1}=-\sin\left(\theta_{0}\right)\theta_{1},\\ A_{2}=-\cos\left(\theta_{0}\right)\theta_{1}^{2}-\sin\left(\theta_{0}\right)\theta_{2},\\ \vdots \end{array}$$ (16)

so the first few terms of our decomposition series are

$$\begin{array}{c} \theta_{1}=2\eta t,\\ \theta_{2}=0,\\ \theta_{3}=\frac{2}{3}\left(\eta^{2}-\eta^{3}\right)t^{3},\\ \vdots \end{array}$$ (17)

thus, we can see emerging the Taylor series representation of the *a priori* known solution $2\;\arctan\left(\sqrt{\eta }\tan\left(\sqrt{\eta }t\right)\right.$, which is expressed as^37^

$$\theta \left(t\right)=2\left(\overset{\infty }{\underset{j=0}{\sum }}\left(\frac{{\left(-1\right)}^{j}}{2j+1}{\left(\sqrt{\eta }\overset{\infty }{\underset{k=1}{\sum }}\left(\frac{{\left(-1\right)}^{k-1}\left(2^{2k}-1\right)2^{2k}B_{2k}}{\left(2k\right)!}{\left(\sqrt{\eta }t\right)}^{2k-1}\right)\right)}^{2j+1}\right)\right),$$ (18)

where *B* are the Bernoulli numbers.^38^

### B. FitzHugh–Nagumo model

First devised as an oscillator^10^ and then as an equivalent circuit,^11^ the Fitzhugh–Nagumo model consists of two coupled nonlinear differential equations given as^39^

$$\frac{dV}{dt}=V-\frac{V^{3}}{3}-W+\sigma ,$$ (19)

$$\frac{dW}{dt}=\phi \left(V+\alpha -\beta W\right),$$ (20)

where *V* is the transmembrane potential, *W* is the recovery variable, *σ* is the stimulating current, and *ϕ*, *β*, and *α* are positive constants. The operators of the *V* Eq. (19) are $LV=\frac{dV}{dt}$, R{V,W}=V−W, $NV=V^{3}/3$, and *g*(*t*) = *σ* while the *W* Eq. (20) has $LW=\frac{dW}{dt}$, R{V,W}=ϕ(V−βW), *g*(*t*) = *ϕα*, and no N term. Let $V\left(t\right)=\sum_{n=0}^{\infty }V_{n}$, $W\left(t\right)=\sum_{n=0}^{\infty }W_{n}$, and $NV=\frac{1}{3}\sum_{n=0}^{\infty }A_{n}\left(V^{3}\right)$. When we apply $L^{-1}$ to Eqs. (19) and (20) we respectively get

$$V\left(t\right)=V\left(0\right)+\sigma t+L^{-1}\left\{\overset{\infty }{\underset{n=0}{\sum }}\left(V_{n}-\frac{A_{n}\left(V^{3}\right)}{3}-W_{n}\right)\right\},$$ (21)

$$W\left(t\right)=W\left(0\right)+\phi \alpha t+\phi L^{-1}\left\{\overset{\infty }{\underset{n=0}{\sum }}\left(V_{n}-\beta W_{n}\right)\right\}.$$ (22)

The recursion schemes are

$$\begin{array}{c} V_{0}=V\left(0\right),\\ V_{1}=\sigma t+L^{-1}\left\{V_{0}-\frac{A_{0}\left(V^{3}\right)}{3}-W_{0}\right\},\\ V_{n+2}=L^{-1}\left\{V_{n+1}-\frac{A_{n+1}\left(V^{3}\right)}{3}-W_{n+1}\right\}, \end{array}$$ (23)

and

$$\begin{array}{c} W_{0}=W\left(0\right),\\ W_{1}=\phi \alpha t+\phi L^{-1}\left\{V_{0}-\beta W_{0}\right\},\\ W_{n+2}=\phi L^{-1}\left\{V_{n+1}-\beta W_{n+1}\right\}. \end{array}$$ (24)

However, for a few highly restricted cases, e.g., Demina and Kudryashov’s meromorphic,^40^ we have no *a priori* solution, so we must verify our results with the error $e=F\left\{\varphi_{m}\left(t\right)\right\}-g\left(t\right)$, which comes from Eq. (1). If we write our *m*-term partial sums as $v_{m}\left(t\right)=\sum_{n=0}^{m-1}V_{n}\left(t\right)$ and $w_{m}\left(t\right)=\sum_{n=0}^{m-1}W_{n}\left(t\right)$, then the infinity norm of the error ‖*e*‖_∞_ from the *V* Eq. (19) and the *W* Eq. (20) (denoted by subscript) are, respectively, given as

$$\|e_{v}{\|}_{\infty }=\underset{t_{k}\leq t\leq t_{k+1}}{\max}\left|\frac{dv_{m}\left(t\right)}{dt}-v_{m}\left(t\right)+\frac{v_{m}{\left(t\right)}^{3}}{3}+w_{m}\left(t\right)-\sigma \right|,$$ (25)

$$\|e_{w}{\|}_{\infty }=\underset{t_{k}\leq t\leq t_{k+1}}{\max}\left|\frac{dw_{m}\left(t\right)}{dt}-\phi \left(v_{m}\left(t\right)+\alpha -\beta w_{m}\left(t\right)\right)\right|.$$ (26)

**FIG. 1.**
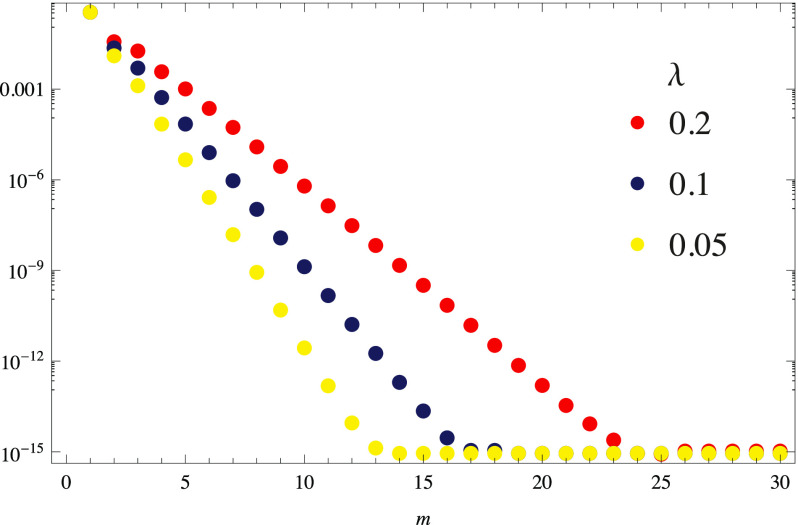
Maximum error from Eq. (25) as a function of *m* over the interval size *h*_0_ = *λr*^(0)^ where *r*^(0)^ = 1.8.

Figure 1 shows the ‖*e*_*v*_‖_∞_ for the primitive element *k* = 0 whose *r*^(0)^ = 1.8 for *λ* = 0.05, 0.1, and 0.2, and as expected, our solution converges more quickly as *λ* is decreased. Note that, by inspection, we can see that the error decreases linearly, which means that, since this is a logarithmic scale, the rates of convergence are exponential. The ‖*e*_*w*_‖_∞_ (not shown) looks the same as ‖*e*_*v*_‖_∞_ for all intervals, which is expected since their interval length *h*_*k*_ corresponds to the local *r*^(*k*)^.

**FIG. 2.**
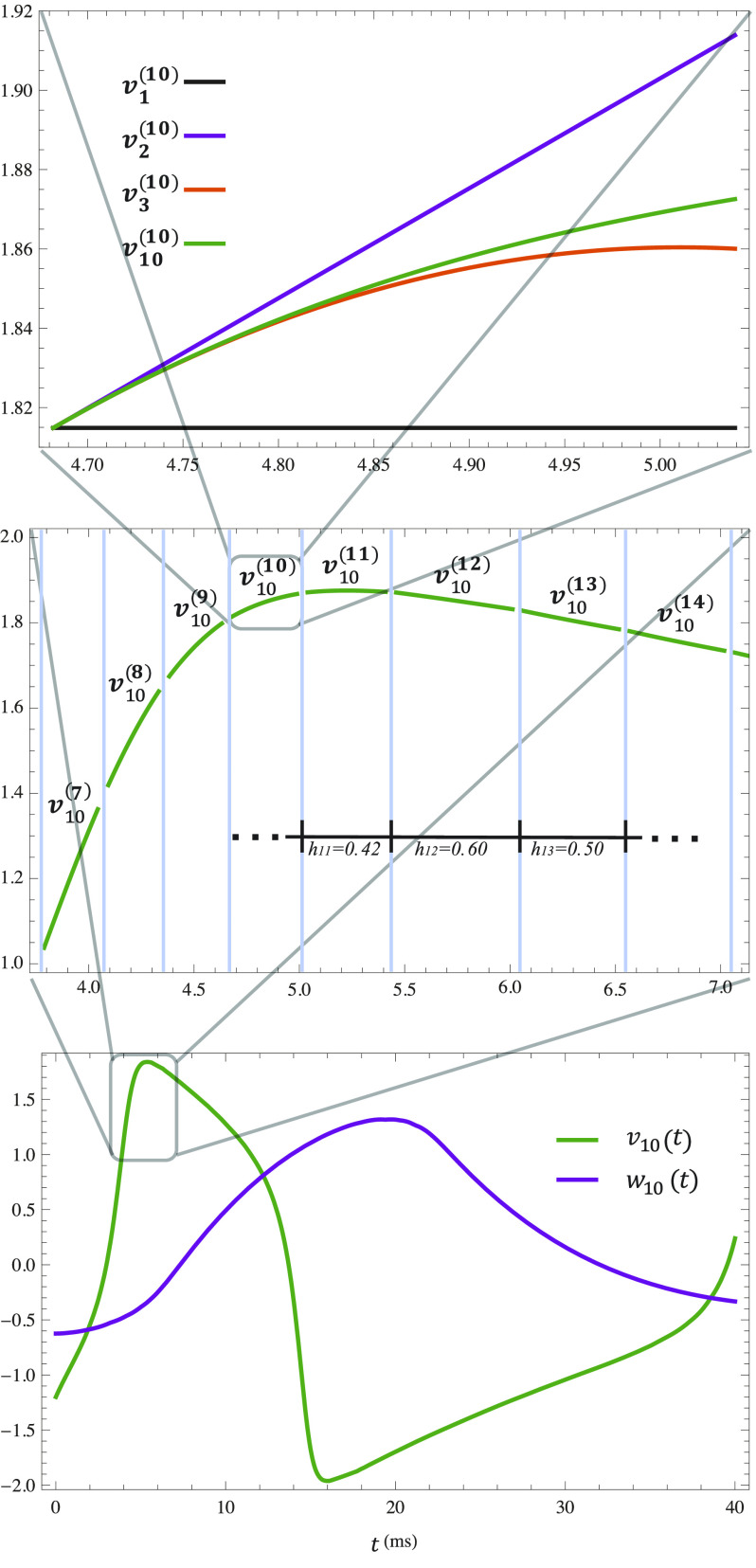
(Bottom) The splines of *v*_10_(*t*) and *w*_10_(*t*) over the course of one action potential. The modeling inputs are listed in Table I. (Middle) A zoomed in view of *v*_10_(*t*) where the light blue vertical lines separate the spline’s individual elements. A few of the intervals are labeled. (Top) A zoomed in view of $v_{10}^{\left(10\right)}\left(t\right)$ where 1, 2, and 3 term partial sum solutions are also plotted. The vertical axes’ units are mV.

Let us now use our solution to construct splines (shown in Fig. 2) depicting an action potential using *v*_10_(*t*) and *w*_10_(*t*) and varying the interval length. We take the inputs (listed in Table I) from FitzHugh’s work.^39^ The bottom panel shows the full dynamic behavior of the two state variables. Right above it, we zoom into a sample of the *v*_10_(*t*) spline to show the varying interval lengths. Above that, we zoom in even further to focus on just one element $v_{10}^{\left(10\right)}\left(t\right)$ to show how quickly the curve converges as we add terms.

**TABLE I.** FitzHugh–Nagumo modeling inputs.

Parameter	Value
Stimulating current, *σ*	0.35
Interval dilate, *λ*	0.25
Solution order, *m*	10
Tunable constants	
*α*	0.7
*β*	0.8
*ϕ*	0.08
Initial conditions	
${\left.V\left(t\right)\right|}_{t=0}$	−1.1994
${\left.W\left(t\right)\right|}_{t=0}$	−0.6243

### C. Hindmarsh–Rose model

Hindmarsh and Rose first modified the two state variable FitzHugh–Nagumo model of action potentials to allow for long interspike intervals to resemble the behavior of real neurons more closely.^41^ Later they added a third state variable, which allows for a qualitative description of bursting behavior,^12^ which we now consider. The model is given as

$$\frac{dX}{dt}=Y-aX^{3}+bX^{2}-Z+I,$$ (27)

$$\frac{dY}{dt}=c-dX^{2}-Y,$$ (28)

$$\frac{dZ}{dt}=r\left(s\left(X-X_{R}\right)-Z\right),$$ (29)

where *X* is the transmembrane potential, *Y* reflects the spiking Na^+^ and K^+^ currents, *Z* is an adaptive current that terminates them, thus creating an isolated burst of spikes, *I* is the stimulating current, and *a*, *b*, *c*, *d*, *r*, *s*, and *X*_*R*_ are constants. In all three equations of Eq. (24), we recognize $L=\frac{d}{dt}$, and thus, $L^{-1}=\int_{0}^{t}dt$. We decompose the variables $X\left(t\right)=\sum_{n=0}^{\infty }X_{n}$, $Y\left(t\right)=\sum_{n=0}^{\infty }Y_{n}$, and $Z\left(t\right)=\sum_{n=0}^{\infty }Z_{n}$ as well as the nonlinear terms in Eq. (27) $NX=\sum_{n=0}^{\infty }\left(A_{n}\left(X^{3}\right)+A_{n}\left(X^{2}\right)\right)$ and Eq. (28) $NX=\sum_{n=0}^{\infty }A_{n}\left(X^{2}\right)$, and we apply $L^{-1}$ throughout Eq. (24) to obtain

$$\begin{array}{ll} X\left(t\right) & =\overset{\infty }{\underset{n=0}{\sum }}X_{n}=X\left(0\right)+It\\ & \;+\;L^{-1}\left\{\overset{\infty }{\underset{n=0}{\sum }}\left(y_{n}-aA_{n}\left(X^{3}\right)+bA_{n}\left(X^{2}\right)-Z_{n}\right)\right\}, \end{array}$$ (30)

$$Y\left(t\right)=\overset{\infty }{\underset{n=0}{\sum }}Y_{n}=Y\left(0\right)+ct-L^{-1}\left\{\overset{\infty }{\underset{n=0}{\sum }}\left(dA_{n}\left(X^{2}\right)+Y_{n}\right)\right\},$$ (31)

$$Z\left(t\right)=\overset{\infty }{\underset{n=0}{\sum }}Z_{n}=Z\left(0\right)-rsX_{R}t+L^{-1}\left\{\overset{\infty }{\underset{n=0}{\sum }}\left(rsX_{n}-rZ_{n}\right)\right\}.$$ (32)

The recursion schemes are

$$\begin{array}{c} X_{0}=X\left(0\right),\\ X_{1}=It+L^{-1}\left\{Y_{0}-aA_{0}\left(X^{3}\right)+bA_{0}\left(X^{2}\right)-Z_{0}\right\},\\ X_{n+2}=L^{-1}\left\{Y_{n+1}-aA_{n+1}\left(X^{3}\right)+bA_{n+1}\left(X^{2}\right)-Z_{n+1}\right\}, \end{array}$$ (33)

$$\begin{array}{c} Y_{0}=Y\left(0\right),\\ Y_{1}=ct-L^{-1}\left\{dA_{0}\left(X^{2}\right)+Y_{0}\right\},\\ Y_{n+2}=L^{-1}\left\{dA_{n+1}\left(X^{2}\right)+Y_{n+1}\right\}, \end{array}$$ (34)

and

$$\begin{array}{c} Z_{0}=Z\left(0\right),\\ Z_{1}=-rsX_{R}t+L^{-1}\left\{rsX_{0}-rZ_{0}\right\},\\ Z_{n+2}=L^{-1}\left\{rsX_{n+1}-rZ_{n+1}\right\}. \end{array}$$ (35)

Writing our *m*-term partial sums as $x_{m}\left(t\right)=\sum_{n=0}^{m-1}X_{n}\left(t\right)$, $y_{m}\left(t\right)=\sum_{n=0}^{m-1}Y_{n}\left(t\right)$, and $z_{m}\left(t\right)=\sum_{n=0}^{m-1}Z_{n}\left(t\right)$ means our ‖*e*‖_∞_ values are written as

$$\begin{array}{ll} \|e_{x}{\|}_{\infty } & =\underset{t_{k}\leq t\leq t_{k+1}}{max}\left|\begin{array}{c} \frac{dx_{m}\left(t\right)}{dt}-y_{m}\left(t\right)+ax_{m}^{3}\left(t\right)\\ \;-bx_{m}^{2}\left(t\right)+z_{m}\left(t\right)-I \end{array}\right| \end{array}\;\;$$ (36)

$$\|e_{y}{\|}_{\infty }=\underset{t_{k}\leq t\leq t_{k+1}}{\max}\left|\frac{dy_{m}\left(t\right)}{dt}-c+dx_{m}^{2}\left(t\right)+y_{m}\left(t\right)\right|,$$ (37)

$$\|e_{z}{\|}_{\infty }=\underset{t_{k}\leq t\leq t_{k+1}}{\max}\left|\frac{dz_{m}\left(t\right)}{dt}-r\left(s\left(x_{m}\left(t\right)-X_{R}\right)-z_{m}\left(t\right)\right)\right|.$$ (38)

**FIG. 3.**
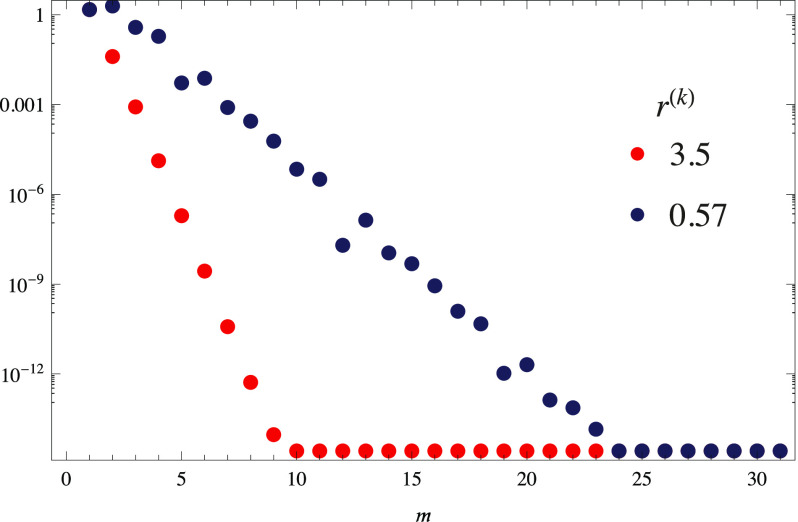
Maximum error for the partial sum solutions from Eq. (36) as a function of *m* over the intervals *k* = 266 and 342 whose respective radii of convergence are 3.5 and 0.57, respectively.

Figure 3 shows the ‖*e*_*x*_‖_*∞*_ at intervals *k* = 266 and 342 whose respective *r*^(*k*)^ = 3.5 and 0.57, respectively. Once again, by inspection, we can recognize exponential convergence rates from the (approximately) linear shape on the log scale. Furthermore, we see a faster convergence in the interval with the smaller radius. The ‖*e*_*y*_‖_*∞*_ and ‖*e*_*z*_‖_*∞*_ are similar (not shown).

**FIG. 4.**
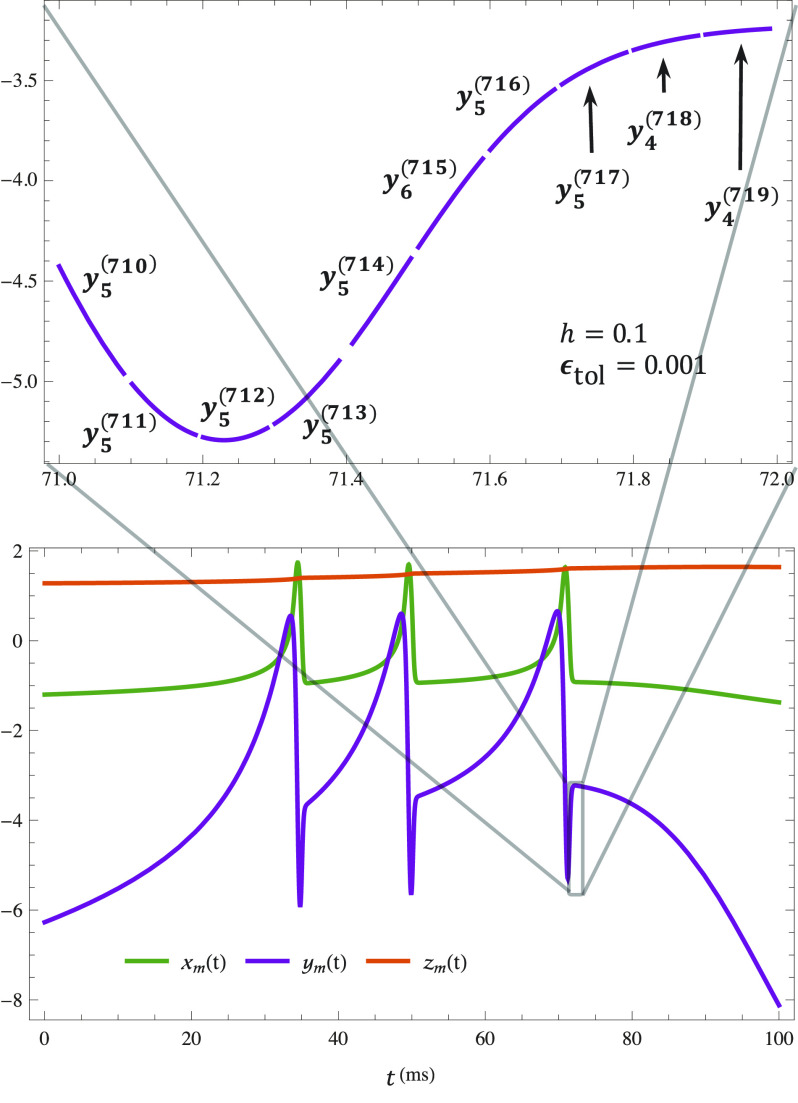
(Bottom) The splines of *x*_*m*_(*t*), *y*_*m*_(*t*), and *z*_*m*_(*t*) over the course of a three spike burst. The spline interval length *h* = 0.1, and the truncation error tolerance is *ɛ*_tol_ = 0.001. (Top) A zoomed in view of *y*_*m*_(*t*). Since the time interval is fixed, each element corresponds to an easily calculated time. The vertical axes’ units are mV.

Using inputs from the work by Hindmarsh and Rose^12^ (listed in Table II), we now construct splines (plotted in Fig. 4) that show a burst of spikes. This time, however, we hold the interval length fixed at *h* = 0.1 and allow the order of each element to vary, maintaining a truncation error at or below a set tolerance of *ɛ*_tol_. In the top panel of Fig. 4, we zoom in to a section of the *y*_*m*_(*t*) spline to see individual elements labeled with their respective orders.

**TABLE II.** Hindmarsh–Rose modeling inputs.

Parameter	Value
Stimulating current, *I*	1.5
Interval length, *h*	0.1
Tolerance, *ɛ*_tol_	0.001
Tunable constants	
*a*	1
*b*	3
*c*	1
*d*	5
*r*	0.0021
*s*	4
*X*_*R*_	$-\frac{8}{5}$
Initial conditions	
${\left.X\left(t\right)\right|}_{t=0}$	−1.200 49
${\left.Y\left(t\right)\right|}_{t=0}$	−6.270 14
${\left.Z\left(t\right)\right|}_{t=0}$	1.277 97

## IV. COMPARISON WITH CARDINAL BASIS SPLINES

### A. Meromorphic FitzHugh–Nagumo model

Having shown how to construct spline solutions with the ADM (A-splines), we now validate this method through comparison to the method of collocation and cardinal basis splines (B-splines). For convenience, we will solve a version of the FitzHugh–Nagumo model (inputs in Table III),

$$\begin{array}{c} \frac{dv}{dt}=v-v^{3}-w+\sigma ,\\ \frac{dw}{dt}=\phi \left(v+\alpha -\beta w\right), \end{array}$$ (39)

for which we have the analytic solutions in closed form,^40^

$$v\left(t\right)=\sqrt{\frac{7}{10}+\frac{1}{5}e^{-\frac{2}{5}t}+\frac{1}{10}\tanh\left(\frac{4}{5}\right)},$$ (40)

$$w\left(t\right)=\frac{4\sqrt{10}+25\sigma \sqrt{7+2e^{-\frac{2}{5}t}+\tanh\left(\frac{4}{5}\right)}-e^{-\frac{4}{5}t}\sqrt{10}}{25\sqrt{7+2e^{-\frac{2}{5}t}+\tanh\left(\frac{4}{5}\right)}}.$$ (41)

**TABLE III.** Meromorphic FitzHugh–Nagumo modeling inputs.

Parameter	Value
Stimulating current, *σ*	0.35
Solution order, *m*	3
Interval, *T*	1
Knot spacing. *δ*	3*h*
Tunable constants	
*α*	*βσ*
*β*	5
*ϕ*	$\frac{3}{25}$
Initial conditions	
${\left.V\left(t\right)\right|}_{t=0}$	$\frac{3}{\sqrt{10}}$
${\left.W\left(t\right)\right|}_{t=0}$	$\frac{7}{20}+\frac{1}{5}\sqrt{\frac{2}{5}}$

### B. B-spline collocation

We adopt the approach given by Pitolli;^42^ however, for a thorough treatment of B-spline theory and applications, please see the works by Schumaker^43^ and Prenter.^44^ Briefly, we partition △ the time interval [0, *T*] into uniformly spaced integers *l* (called knots) as △_*δ*_ ≔ {*lδ*, 0 ≤ *l* ≤ *N*}, where $N=\frac{T}{\delta }$ and *δ* is the dilate for the knot time interval. We use spline curves for our approximating functions,

$$\begin{array}{c} v_{\delta }\left(t\right)=\overset{N}{\underset{l=0}{\sum }}\nu_{l}B_{l}^{m}\left(t\right),\\ w_{\delta }\left(t\right)=\overset{N}{\underset{l=0}{\sum }}\omega_{l}B_{l}^{m}\left(t\right), \end{array}$$ (42)

where *ν*_*l*_ and *ω*_*l*_ are unknown coefficients and $B_{l}^{m}\left(t\right)$ are the B-spline functions, piecewise polynomials of order *m* interleaved at the knots, recursively defined as^45^

$$B_{l}^{0}\left(t\right)=\left\{\begin{array}{cc} 1, & t_{l}\leq t\leq t_{l+1},\\ 0, & otherwise, \end{array}\right.\;m=0,$$ (43)

$$B_{l}^{m}\left(t\right)=\frac{t-t_{l}}{t_{l+m}-t_{l}}B_{l}^{m-1}\left(t\right)+\frac{t_{l+m+1}-t}{t_{l+m+1}-t_{l+1}}B_{l+1}^{m-1}\left(t\right),m\geq 1.$$ (44)

Our knot vector is equally spaced with *t* = 0 having multiplicity *m* + 1.^46^ The solution then involves solving for *ν*_*l*_ and *ω*_*l*_ through the method of collocation. We do this by substituting Eq. (42) into Eq. (39) to get

$$\begin{array}{c} \nu_{l}\frac{d}{dt}B_{l}^{m}\left(t\right)=\nu_{l}B_{l}^{m}\left(t\right)-{\left(\nu_{l}B_{l}^{m}\left(t\right)\right)}^{3}-\omega_{l}B_{l}^{m}\left(t\right)+\sigma ,\\ \omega_{l}\frac{d}{dt}B_{l}^{m}\left(t\right)=\phi \left(\nu_{l}B_{l}^{m}\left(t\right)-\beta \omega_{l}B_{l}^{m}\left(t\right)\right)+\phi \alpha , \end{array}$$ (45)

(summation over *l* ∈ [0, *N*] is understood) and evaluating our B-splines at collocation points, which are expressed as △_*τ*_ ≔ {*t*_*p*_ = *pτ*, 0 ≤ *p* ≤ *P*}, where $P=\frac{T}{\tau }\leq N$. We solve this nonlinear system of 2(*P* + 1) equations through 2(*m* + *N*) unknowns by the Gauss–Newton method on Mathematica 13.1 (Wolfram Research, Inc., Champaign, IL). A detailed description of the method can be found in Ref. 42.

### C. Example results

For our numeric example, we look at a small interval [0, 1] and use cubic splines, i.e., *m* = 3. All inputs are summarized in Table III. In this example, we want to show that the ADM is at least as accurate as the B-spline collocation method by comparing the infinity norm of the errors, which we define as *e*_*B*_ = *v* − *v*_*δ*_ and *e*_*A*_ = *v* − *v*_*m*_ at various discretizations $h=\frac{1}{3}\delta$. We list in Table IV the errors for an increasingly fine mesh. As expected, when the error goes down, the time steps are shorter. At all levels, the A-spline is around an order of magnitude more accurate than the B-spline approximation.

**TABLE IV.** The maximum error for the B and A splines.

*h*	‖*e*_*B*_‖_∞_	‖*e*_*A*_‖_∞_
$\frac{1}{6}$	3.7751 × 10^−6^	5.0039 × 10^−7^
$\frac{1}{12}$	3.3928 × 10^−7^	6.8181 × 10^−8^
$\frac{1}{24}$	2.3888 × 10^−8^	8.8124 × 10^−9^
$\frac{1}{48}$	1.5699 × 10^−9^	1.1175 × 10^−9^

Finally, as a further check of the ADM, following the same steps in Sec. III B but this time with the meromorphic formulation and inputs, the first few terms of our decomposition series are

$$\begin{array}{c} v_{m}\left(t\right)=\frac{3}{\sqrt{10}}-\frac{t}{10\sqrt{10}}+\frac{t^{2}}{40\sqrt{10}}+\cdots \;,\\ w_{m}\left(t\right)=\frac{35}{100}-\frac{4}{\sqrt{10}}+\frac{3t}{25\sqrt{10}}-\frac{21t^{2}}{500\sqrt{10}}+\cdots \;, \end{array}$$ (46)

revealing the Taylor series expansions for our known solutions.

## V. CONCLUSION

The solutions we presented here represent a new way for neural engineers to approach key biophysical models. They are just a tiny fraction of the analytic possibilities with the ADM. For instance, besides analytic continuation, the solutions’ validity can also be expanded through, e.g., diagonal Padé approximates^47^ or iterated Shanks transforms.^48^ For the purposes of MREIT, generalizations worth considering include partial differential equations^49^ for impulse propagation, state variables describing multi-physics phenomena like the neural bilayer sonophore,^50^ and arbitrary derivative order^51^ to model non-ohmic conductivities in tissue.^52^

## Data Availability

The data that support the findings of this study are available within the article.

## APPENDIX A: MATHEMATICA CODE FOR ADOMIAN POLYNOMIALS

Here now is some of our Mathematica codes written in the Wolfram language, which allows for 2D input, Greek letters, and symbolic operations. First is the code for calculating the Adomian polynomials defined by Eq. (5) for $Nv=v^{3}$.

AP[m_]≔ Module[{F}, (*define the operation*)

f[*v*_]≔ *v*^3^;

F_0_ = f$\left[\sum_{k=0}^{m}\left(v_{m-1,k}\left[t\right]S^{k}\right)\right]$;

For $\left[i\right.$ = 0, *i* ≤ *m*, *i* + +,

A[i] = Expand[F_*i*_/.S → 0];

F_*i*+1_ = $\frac{1}{i+1}$D[F_*i*_,S]]]

AP[n] (*calculate n polynomials*)

## APPENDIX B: MATHEMATICA CODE FOR A-SPLINE

Finally, we present our code for the elements of a basic A-spline solution for the FitzHugh–Nagumo model given in Eqs. (19) and (20). For simplicity, the *K* intervals each have a constant duration *h* = *T* and polynomial order *m* = *M*.

*v*_0,0_[*t*_] = −1.199 41; (*first terms from initial conditions*)

*w*_0,0_[*t*_] = −0.6243;

Fork = 1, *k* ≤ *K*, *k* = *k*+1, (*intervals loop*)

*v*_*k*−1,1_[*t*_] = *σ t*+$\int_{0}^{t}\left(v_{k-1,0}\left[t\right]\right.$–$\frac{1}{3}A\left[0\right]$–$\left.w_{k-1,0}\left[t\right]\right)dt$;

*w*_*k*−1,1_[*t*_] = *ϕαt*+$\phi \left(\int_{0}^{t}\left(v_{k-1,0}\left[t\right]\right.\right.$–$\left.\left.\beta w_{k-1,0}\left[t\right]\right)dt\right)$;

For $\left[m\right.$ = 1, *m* ≤ *M*, *m* = *m* + 1, (*series loop*)

*v*_*k*−1,*m*_[*t*_] = $\int_{0}^{t}\left(v_{k-1,m-1}\left[t\right]\right.$–$\frac{1}{3}A\left[m\right.$–1]−$\left.w_{k-1,m-1}\left[t\right]\right)dt$;

*w*_*k*−1,*m*_[*t*_] = $\phi \left(\int_{0}^{t}\left(v_{k-1,m-1}\left[t\right]\right.\right.$–$\left.\left.\beta w_{k-1,m-1}\left[t\right]\right)dt\right)$;]

*v*_*k*,0_[*t*_] = $\sum_{m=0}^{M}\left(v_{k-1,m}\left[T\right]\right)$; (*end of each interval*)

*w*_*k*,0_[*t*_] = $\sum_{m=0}^{M}\left(w_{k-1,m}\left[T\right]\right)$;]

## References

[1] D. T. Wymer, K. P. Patel, W. F. Burke III, and V. K. Bhatia, “Phase-contrast MRI: Physics, techniques, and clinical applications,” Radiographics 40, 122–140 (2020). 10.1148/rg.2020190039 31917664

[2] M. C. Murphy, J. Huston III, and R. L. Ehman, “MR elastography of the brain and its application in neurological diseases,” Neuroimage 187, 176–183 (2019). 10.1016/j.neuroimage.2017.10.008 28993232 PMC5889749

[3] J. K. Seo and E. J. Woo, “Magnetic resonance electrical impedance tomography (MREIT),” SIAM Rev. 53, 40–68 (2011). 10.1137/080742932

[4] R. J. Sadleir, S. C. Grant, and E. J. Woo, “Can high-field MREIT be used to directly detect neural activity? Theoretical considerations,” NeuroImage 52, 205–216 (2010). 10.1016/j.neuroimage.2010.04.005 20382240 PMC2903888

[5] R. J. Sadleir, F. Fu, C. Falgas, S. Holland, M. Boggess, S. C. Grant, and E. J. Woo, “Direct detection of neural activity in vitro using magnetic resonance electrical impedance tomography (MREIT),” NeuroImage 161, 104–119 (2017). 10.1016/j.neuroimage.2017.08.004 28818695 PMC5696120

[6] W. Rall and H. Agmon-Snir, “Cable theory for dendritic neurons,” in Methods in Neuronal Modeling: From Ions to Networks, 2nd ed., edited by C. Koch and I. Segev (MIT Press, Cambridge, 1988), pp. 27–92.

[7] B. L. Schwartz, M. Chauhan, and R. J. Sadleir, “Analytic modeling of neural tissue: I. A spherical bidomain,” J. Math. Neurosci. 6, 1–20 (2016). 10.1186/s13408-016-0041-1 27613652 PMC5018001

[8] G. B. Ermentrout and N. Kopell, “Parabolic bursting in an excitable system coupled with a slow oscillation,” SIAM J. Appl. Math. 46, 233–253 (1986). 10.1137/0146017

[9] N. Kopell and G. B. Ermentrout, “Subcellular oscillations and bursting,” Math. Biosci. 78, 265–291 (1986). 10.1016/0025-5564(86)90128-8

[10] R. Fitzhugh, “Impulses and physiological states in theoretical models of nerve membrane,” Biophys. J. 1, 445–466 (1961). 10.1016/s0006-3495(61)86902-6 19431309 PMC1366333

[11] J. Nagumo, S. Arimoto, and S. Yoshizawa, “An active pulse transmission line simulating nerve axon,” Proc. IRE 50, 2061–2070 (1962). 10.1109/jrproc.1962.288235

[12] J. L. Hindmarsh and R. M. Rose, “A model of neuronal bursting using three coupled first order differential equations,” Proc. R. Soc. B 221, 87–102 (1984). 10.1098/rspb.1984.0024 6144106

[13] T. Öziş and A. Yıldırım, “Comparison between Adomian’s method and He’s homotopy perturbation method,” Comput. Math. Appl. 56, 1216–1224 (2008). 10.1016/j.camwa.2008.02.023

[14] G. Adomian, Solving Frontier Problems of Physics: The Decomposition Method (Klywer Academic Publishers, Norwell, MA, 1994), pp. 6–20.

[15] G. Adomian, “A review of the decomposition method and some recent results for nonlinear equations,” Comput. Math. Appl. 21, 101–127 (1991). 10.1016/0898-1221(91)90220-x

[16] R. Rach, “A bibliography of the theory and applications of the Adomian decomposition method, 1961–2011,” Kybernetes 41, 1087–1148 (2012). 10.1108/k.2012.06741gaa.007

[17] G. Adomian, G. E. Adomian, and R. E. Bellman, “Biological system interactions,” Proc. Natl. Acad. Sci. U. S. A. 81, 2938–2940 (1984). 10.1073/pnas.81.9.2938 6585837 PMC345189

[18] G. Adomian and G. E. Adomian, “Cellular systems and aging models,” Comput. Math. Appl. 11, 283–291 (1985). 10.1016/0898-1221(85)90153-1

[19] G. Adomian and G. E. Adomian, “Solution of the Marchuk model of infections disease and immune response,” Math. Modell. 7, 803–807 (1986). 10.1016/0270-0255(86)90136-3

[20] G. Adomian, “Solving the mathematical models of neurosciences and medicine,” Math. Comput. Simul. 40, 107–114 (1995). 10.1016/0378-4754(95)00021-8

[21] G. Adomian, M. Witten, and G. E. Adomian, “A new approach to the solution of neurological models: Application to the Hodgkin Huxley and the FitzHugh Nagumo equations,” in Stochastic Processes and Their Applications, edited by M. J. Beckmann, M. N. Gopalan, and R. Subramanian (Springer, Berlin, 1991), pp. 99–113.

[22] R. Rach, “A convenient computational form for the Adomian polynomials,” J. Math. Anal. Appl. 102, 415–419 (1984). 10.1016/0022-247x(84)90181-1

[23] A.-M. Wazwaz, “A reliable modification of Adomian decomposition method,” Appl. Math. Comput. 102, 77–86 (1999). 10.1016/s0096-3003(98)10024-3

[24] Y. Cherruault and G. Adomian, “Decomposition methods: A new proof of convergence,” Math. Comput. Modell. 18, 103–106 (1993). 10.1016/0895-7177(93)90233-o

[25] P. Dennery and A. Krzywicki, Mathematics for Physicists (Dover Publications, Inc., Mineola, NY, 1996), pp. 45–47.

[26] Y. Cherruault, G. Adomian, K. Abbaoui, and R. Rach, “Further remarks on convergence of decomposition method,” Int. J. BioMed. Comput. 38, 89–93 (1995). 10.1016/0020-7101(94)01042-y 7705918

[27] A. Rèpaci, “Nonlinear dynamical systems: On the accuracy of Adomian’s decomposition method,” Appl. Math. Lett. 3, 35–39 (1990). 10.1016/0893-9659(90)90042-a

[28] G. Adomian, R. Rach, and R. Meyers, “Numerical algorithms and decomposition,” Comput. Math. Appl. 22, 57–61 (1991). 10.1016/0898-1221(91)90013-t

[29] G. Adomian, R. C. Rach, and R. E. Meyers, “Numerical integration, analytic continuation, and decomposition,” Appl. Math. Comput. 88, 95–116 (1997). 10.1016/s0096-3003(96)00052-5

[30] J.-S. Duan, R. Rach, A.-M. Wazwaz, T. Chaolu, and Z. Wang, “A new modified Adomian decomposition method and its multistage form for solving nonlinear boundary value problems with Robin boundary conditions,” Appl. Math. Modell. 37, 8687–8708 (2013). 10.1016/j.apm.2013.02.002

[31] A. Ben-Menahem and S. J. Singh, Seismic Waves and Sources (Springer-Verlag, New York, NY, 1981), pp. 44–88.

[32] W. Gellert, H. Küstner, M. Hellwich, and H. Kästner, The VNR Concise Encyclopedia of Mathematics (Van Nostrand Reinhold Company, New York, NY, 1975), pp. 483–484.

[33] J.-S. Duan and R. Rach, “New higher-order numerical one-step methods based on the Adomian and modified decomposition methods,” Appl. Math. Comput. 218, 2810–2828 (2011). 10.1016/j.amc.2011.08.024

[34] B. Ermentrout, “Type I membranes, phase resetting curves, and synchrony,” Neural Comput. 8, 979–1001 (1996). 10.1162/neco.1996.8.5.979 8697231

[35] C. Börgers and N. Kopell, “Synchronization in networks of excitatory and inhibitory neurons with sparse, random connectivity,” Neural Comput. 15, 509–538 (2003). 10.1162/089976603321192059 12620157

[36] B. S. Gutkin and G. B. Ermentrout, “Dynamics of membrane excitability determine interspike interval variability: A link between spike generation mechanisms and cortical spike train statistics,” Neural Comput. 10, 1047–1065 (1998). 10.1162/089976698300017331 9654767

[37] J. Spanier and K. B. Oldham, An Atlas of Functions (Hemisphere Publishing Corporation, New York, NY, 1987), pp. 319–341.

[38] R. W. Hamming, Numerical Methods for Scientists and Engineers (Dover Publications, Inc., Mineola, NY, 1986), pp. 189–191.

[39] R. FitzHugh, “Mathematical models of excitation and propagation in nerve,” in Biological Engineering, edited by H. P. Schwan (McGraw-Hill Book Company, New York, NY, 1969), pp. 1–85.

[40] M. V. Demina and N. A. Kudryashov, “Meromorphic solutions in the FitzHugh–Nagumo model,” Appl. Math. Lett. 82, 18–23 (2018). 10.1016/j.aml.2018.02.012

[41] J. L. Hindmarsh and R. M. Rose, “A model of the nerve impulse using two first-order differential equations,” Nature 296, 162–164 (1982). 10.1038/296162a0 7063018

[42] F. Pitolli, “A collocation method for the numerical solution of nonlinear fractional dynamical systems,” Algorithms 12, 156 (2019). 10.3390/a12080156

[43] L. L. Schumaker, Spline Functions: Basic Theory (John Wiley & Sons, New York, 1981).

[44] P. M. Prenter, Splines and Variational Methods (John Wiley & Sons, New York, 1975).

[45] C. deBoor, A Practical Guide to Splines, revised ed. (Springer-Verlag, New York, 2001).

[46] F. Pitolli, “Optimal B-spline bases for the numerical solution of fractional differential problems,” Axioms 7, 46 (2018). 10.3390/axioms7030046

[47] M. Dehghan, A. Hamidi, and M. Shakourifar, “The solution of coupled Burgers’ equations using Adomian–Pade technique,” Appl. Math. Comput. 189, 1034–1047 (2007). 10.1016/j.amc.2006.11.179

[48] S. K. Asok, “An application of the Adomian decomposition method to the transient behavior of a model biochemical reaction,” J. Math. Anal. Appl. 132, 232–245 (1988). 10.1016/0022-247X(88)90202-8

[49] G. Adomian and R. Rach, “Equality of partial solutions in the decomposition method for linear or nonlinear partial differential equations,” Comput. Math. Appl. 19, 9–12 (1990). 10.1016/0898-1221(90)90246-g

[50] M. Plaksin, S. Shoham, and E. Kimmel, “Intramembrane cavitation as a predictive bio-piezoelectric mechanism for ultrasonic brain stimulation,” Phys. Rev. X 4, 011004 (2014). 10.1103/physrevx.4.011004

[51] J.-S. Duan, T. Chaolu, R. Rach, and L. Lu, “The Adomian decomposition method with convergence acceleration techniques for nonlinear fractional differential equations,” Comput. Math. Appl. 66, 728–736 (2013). 10.1016/j.camwa.2013.01.019

[52] A. Bueno-Orovio, D. Kay, V. Grau, B. Rodriguez, and K. Burrage, “Fractional diffusion models of cardiac electrical propagation: Role of structural heterogeneity in dispersion of repolarization,” J. R. Soc., Interface 11, 20140352 (2014). 10.1098/rsif.2014.0352 24920109 PMC4208367

